# RACIAL DIFFERENCES IN THE ASSOCIATIONS BETWEEN SENSORY FUNCTIONS AND PHYSICAL ACTIVITY INTENSITY

**DOI:** 10.1093/geroni/igad104.0356

**Published:** 2023-12-21

**Authors:** Qun Le, Linda Churchill, Elizabeth Procter-Gray, Anthony Clarke, Jie Cheng, Danielle LoPilato, Lingming Chen, Wenjun Li

**Affiliations:** University of Massachusetts Lowell, Lowell, Massachusetts, United States; University of Massachusetts, Lowell, Lowell, Massachusetts, United States; University of Massachusetts Lowell, Lowell, Massachusetts, United States; University of Massachusetts Lowell, Lowell, Massachusetts, United States; University of Massachusetts Lowell, Lowell, Massachusetts, United States; University of Massachusetts Lowell, Lowell, Massachusetts, United States; University of Massachusetts Lowell, Lowell, Massachusetts, United States; University of Massachusetts Lowell, Lowell, Massachusetts, United States

## Abstract

Sensory functions decline in older age. Multisensory training has been shown to improve balance. We analyzed the race differences in the associations of sensory functions with physical activity (PA) intensity. The Healthy Aging and Neighborhood Study enrolled 379 community-dwelling persons aged 65 and over in Central Massachusetts (2018-2019). Data on sensory functions were assessed using self-report questionnaires, range from 0 (very poor) to 4 (very good). PA intensities were measured using waist-worn Actigraphy accelerometers for at least 8 hours per day and at least 5 days including 1 weekend day. PA intensity was categorized using Copeland cutoff points as sedentary (SB), light (LPA), moderate-to-vigorous-intensity PA (MVPA). Generalized linear models were used to assess the racial differences in the associations of sensory functions with PA intensity, adjusting for age, gender, education, income, general health, comorbidities, activities of daily living (ADL), and instrumental ADL. Average sensory functions of the participants were good to very good (mean±SD: hearing:2.9±1.1, vision:3.4±0.8, touch:3.7±0.6, smell:3.5±0.9, taste:3.6±0.7, balance:3.0±1.0). Two-thirds (66.7%) of participants met the PA guidelines of MVPA (mins/day) SB:465±114, LPA:248±66, MVPA:53±70). Compared to other races, Whites with better touch (b=52.1, SE=23.8) had significantly more SB. Better hearing (b=16.7, SE=7.2), vision (b=32.1, SE=10.5), smell (b=19.3, SE=7.8), taste (b=25.3, SE=9.9) were significantly associated with more LPA. Better vision (RR=1.6, 95%CI:1.1-2.4) and worse touch (RR=0.6, 95%CI:0.4-0.9) were significantly associated with more MVPA. Racial differences are significant in the associations between sensory functions and PA intensity among older adults. The underlying mechanisms should be further investigated to inform future interventions.

